# Current Lack of Evidence for an Effect of Physical Activity Intervention Combined with Pharmacological Treatment on Bone Turnover Biomarkers in People with Osteopenia and Osteoporosis: A Systematic Review

**DOI:** 10.3390/jcm10153442

**Published:** 2021-08-03

**Authors:** Sofia Marini, Giuseppe Barone, Alice Masini, Laura Dallolio, Laura Bragonzoni, Yari Longobucco, Francesca Maffei

**Affiliations:** 1 Department of Biomedical and Neuromotor Science, University of Bologna, 40126 Bologna, Italy; sofia.marini2@unibo.it (S.M.); laura.dallolio@unibo.it (L.D.); 2 Department for Life Quality Studies, Campus of Rimini, University of Bologna, 47921 Rimini, Italy; giuseppe.barone8@unibo.it (G.B.); laura.bragonzoni4@unibo.it (L.B.); francesca.maffei@unibo.it (F.M.); 3 Department of Medicine and Surgery, University of Parma, 43126 Parma, Italy; yari.longobucco2@unibo.it

**Keywords:** physical activity, pharmacological treatment, exercise, training, bone biomarkers, osteoporosis, osteopenia

## Abstract

The process of bone loss occurs silently and progressively with age, often appearing as osteopenia or osteoporosis or related fractures. Given the rapid raise in disease burden and socio-economic costs of these conditions worldwide, drug therapy combined with physical activity can be a useful strategy and bone biomarkers, can represent a useful evaluation tool to assess their effects. The objective of this systematic review, conducted according to PRISMA statement, was to investigate the effects of physical activity interventions combined with drug treatments on bone biomarkers in people with osteopenia and osteoporosis. Through PubMed, Cochrane, Cinahl, Embase, Trip, a comprehensive literature search was performed. Each study’s quality was assessed according to the Cochrane risk-of-bias tool. Out of 582 identified articles, 50 full texts were screened. Only one matched the eligibility criteria. The study, scored as high quality, showed, in both experimental and control groups, an increase of CTX and P1NP bone biomarkers, without statistically significant differences. Based on available evidence, no exhaustive conclusion can be drawn. However, this systematic review critically analyses the literature, highlighting the knowledge gap on combined treatments efficacy assessed by bone biomarkers. Moreover, an outlook is provided for the planning of future studies.

## 1. Introduction

Undeniably, a gradual loss of bone mineral density (BMD) can lead to osteopenia and osteoporosis (OP). The World Health Organization (WHO) establish OP as a BMD T-score of −2.5 or lower at any one location or presenting a previous fragility fracture [1,2]. Whereas, according to the WHO criteria for assessment of dual X-ray absorptiometry (DXA) measurements, osteopenia is defined as a BMD between −1.0 and −2.5 SD below that of a “young normal” adult [2,3]. Although the main burden of diseases occurs in post-menopausal women, bone loss represents an inevitable implication of aging in both women and men [4]. The loss of BMD is due to an alteration of bone remodelling cycle. The balance between bone resorption and bone formation is determined by the process carried out by two cell types: osteoclast (responsible for secretion of proteolytic enzymes which digest bone matrix) and osteoblast (responsible for bone matrix synthesis). Generally, the process of bone loss occurs silently and progressively, besides there are no symptoms until the first fracture happens [5]. The risk of fracture increases with decreases in BMD, albeit most of osteoporotic fractures occur in osteopenic patients. Since, even though fracture risk is lower in osteopenia compared to OP, the osteopenic range present a higher number of subjects at risk [6]. For these reasons, prevent the development of OP and related fractures is the main goal of screening and treating for osteopenia. Indeed, valid fracture prevention would have a great influence on patient’s morbidity and a fundamental impact on mortality. Worldwide in 2010, the number of individuals aged ≥50 years at high risk of osteoporotic fracture, was estimated at 158 million additionally, it is expected to double over the next 40 years [7]. It is estimated that 75 million people in Europe, USA and Japan are affected by OP [8]. Given that, the economic burden of OP including long-term fracture care and pharmacological prevention represent an urgent issue worldwide bound to frightfully increase, a reasonable goal of treatment should be focused on reducing fractures [9,10].

In view of this, multiple strategies are generally applied. Certainly, the first line is represented by pharmacological treatments which are prescribed to reduce the risk of fragility fractures. Another strategy can be represented by physical activity (PA), useful in prevention and treatment of bone loss and its consequences.

Many drugs with distinct mechanisms of action have been approved for the prevention and treatment of OP and are effective and available globally. Pharmacological therapy should be initiated also in patients with osteopenia [10]. These treatments must be prescribed in conjunction with calcium and vitamin D supplements, adequate lifestyle changes, appropriate nutrition and physical activity. As showed in Table 1, pharmacological treatments can be divided into two main categories: Anti-resorptive drugs, which reduce bone resorption preserving BMD, and Anabolic drugs aimed to stimulate bone formation, thereby increasing BMD [5,10]. Currently, alendronate, ibandronate, risedronate and zoledronic acid are the most common Bisphosphonates used for the treatment of OP worldwide [11]. Medications can have oral (daily, weekly, monthly dosing) or parental administration, dosing intervals of 6 months or longer, convenient for many patients improving short-term persistence with therapy. Among selective estrogen receptor modulators, raloxifene is widely used due to its favourable overall risk-benefit ratio [12,13], while teriparatide is the first approved anabolic drug for the treatment of OP [10,14].

PA plays a fundamental role in the prevention and treatment of many diseases, including OP [16,17]. Indeed, PA can increase bone health during childhood and adolescence, while in the adulthood attenuate bone loss, improve or preserve muscle mass, strength, and power, overall reducing the risk of falls. However, not all exercise types are effective at attenuating all fracture risk factors [11]. As stated in the new WHO Guidelines on “Physical Activity and Sedentary Behaviour”, there is high certainty evidence that higher levels of PA that combines balance, strength, gait, and functional training (e.g., multicomponent physical activity) are associated with a reduced rate of falls and risk of injury from falls in older adults. Furthermore, there is moderate certainty evidence that programmes involving multiple exercises may have significant effects on bone health and OP prevention [17]. Also, whole-body vibration therapy (WBVT) is a form of passive activity that has demonstrated positive outcomes for a range of musculoskeletal disorders such as OP, although the evidence remains unclear due to the relatively limited and conflicting interventional studies in older adults with OP [18]. Just like an exercise program, WBVT has different applications methods. A review conducted by Weber-Rajek et al. shows that the vibration frequency set on whole-body vibration changes from 12 Hz to 90 Hz between different analysed studies [19]. Moreover, even if evidence suggested that WBVT might be a valid tool to improve the body composition, the results do not show significant differences due to different frequency levels, making it difficult to determine which frequency is most effective [20].

Concerning the effects of exercise on BMD, research showed that free-living PA is associated with significant but modest improvements in BMD and, at least, seems to generate homeostatic influences on BMD during ageing [18]. Indeed, PA that enhances muscle strength also improve bone mass (in terms of BMD and content) and bone strength of the specific bones stressed and may serve as a valuable measure to prevent, slow, or reverse the loss of bone mass [21]. In particular, PA contributes to bone mineralization not only via mechanotransduction but also inducing anabolic vascular effect, overall improving local tissue metabolism [22]. For these reasons, PA interventions are recommended in the prevention and treatment of OP, also to decrease the risk of future bone fractures by being able to increase BMD, during all stages of life [23,24]. The constant and balanced activity of both osteoblasts and osteoclasts plays a crucial role in regulating the bone remodelling process [25]. The structural integrity of bone is maintained throughout life, so that most of the adult skeleton is replaced about every 10 years [5].

At present, the diagnosis of health bone is generally based on quantitative analysis of BMD by DXA. Besides imaging technique, bone turnover biomarkers are measured as a helpful or complementary diagnostic data [26,27]. Currently, biomarkers represent products of bone proteins linked to bone formation and bone resorption, and they can reflect the physiological or pathological status of bone remodelling [26,28,29]. OP is a silent disease characterized by bone fragility; thus, biomarkers have been proposed as indicators of the integrity of the whole skeleton to assess the early diagnosis of OP [26,27,28]. For their ability to provide specific and dynamic indications on bone turn-over metabolism, biomarkers may also be powerful parameters for monitoring the effect of drug therapy in OP patients [26,30]. The benefits of exercise are recognized to reduce the impact of bone ageing, thus PA is considered a non-pharmacologic strategy for OP [31]. In the last decade, some studies have reported the capability of PA to influence the bone biomarker in osteoporotic patients [32,33,34]. Recently, a crisis in the treatment of osteoporosis have been identified by researchers in this field, since both prescription and compliance to pharmacological treatments have decreased in the last years [35]. Similar trends have been observed for non-pharmacological treatment. The critical point lies in the need of finding ways to ensure that patients who need appropriate treatment for OP are not only prescribed effective medications but are also equipped with the information they need to make informed choices regarding their condition [35]. Therefore, multicomponent interventions may represent the key factor to improving prescription and compliance rates for OP management [18].

The recent clinical guidelines for the prevention and treatment of osteopenia and OP stated that a global approach is needed in this scenario [13,36,37]. The basic components of a combined approach are behavioural interventions, PA program, pharmacological treatment and nutritional guidelines in individuals at high risk for fractures [38].

Nowadays, given the global burden of osteopenia and OP consequences, there is a growing interest in the field of combined treatment strategy to prevent and treat people suffering from these conditions. It has been a while since the situation has been assessed in this regard [39]. This would therefore appear to be a good time to take stock of the situation. Current evidence supports the potential for bone turnover markers to provide clinically useful information for OP management [40]. Consequently, a focus to expand knowledge on the application of biomarkers to monitor the efficacy of OP multicomponent interventions is of great scientific interest. Moreover, in spite of the fact that drugs and physical activity efficacy has been widely confirmed, they are generally applied separately, not combined [41].

In such a scenario, the goal of the present systematic review is to investigate and critically analyse, for the first time, the existing evidence on the effects of combined therapeutic strategy based on pharmacological treatment and PA interventions measured by bone biomarkers in people with osteopenia and OP.

## 2. Materials and Methods

### 2.1. Search Strategy and Data Sources

This systematic review was conducted in accordance with PRISMA recommendations and the criteria of the reporting of meta-analysis guidelines [42]. The systematic review’s protocol was registered in the International Prospective Register of Systematic Reviews (PROSPERO; registration Number CRD42021230809 available from: https://www.crd.york.ac.uk/prospero/display_record.php?ID=CRD42021230809 (accessed on 2 August 2021).

The following Patients, Interventions, Comparators and Outcomes (PICO) question was developed, addressing the primary search objective, through the following search terms: (P) Osteoporotic or osteopenic people, aged 45–80+; (I) Physical activity; (C) Standard pharmacological treatment and no exercise intervention; (O) The effect of physical activity interventions combined with pharmacological treatment on bone biomarkers.

A systematic literature search of MEDLINE (PubMed), Embase (Ovid), Cochrane Central Register of Controlled Trials (Central), and CINAHL (EBSCO) up to January 2021 was conducted to identify all published articles about the effect of physical activity combined with pharmacological treatment on bone biomarkers in people with osteopenia and OP.

We searched electronic databases, with a 10-year publication date limit, because we were interested in recent pharmacologic treatments and approaches. The following criteria were used to define our research: we included only Randomized Controlled Trial, Clinical Trial, Clinical Study, Comparative Study and Observational Study, with Full text available and conducted on Human subjects. We defined a range of population aged 80 and over: 80+ years, Middle Aged + Aged: 45+ years, Middle Aged: 45–64 years, Aged: 65+ years.

Search strategies (strings adapted when necessary to fit the specific search requirements of each database) used the following Boolean expression: keywords and terms: ((Osteoporosis OR Osteoporoses OR Osteoporosis Post-Traumatic OR Osteoporosis Post Traumatic OR Post-Traumatic Osteoporoses OR Post-Traumatic Osteoporosis OR Osteoporosis Senile OR Osteoporoses Senile OR Senile Osteoporoses OR Osteoporosis Involutional OR Senile Osteoporosis OR Osteoporosis Age-Related OR Osteoporosis Age Related OR Bone Loss Age-Related OR Age-Related Bone Loss OR Age-Related Bone Losses OR Bone Loss Age Related OR Bone Losses Age-Related OR Age-Related Osteoporosis OR Age Related Osteoporosis OR Age-Related Osteoporoses OR Osteoporoses Age-Related OR Osteopenia OR Osteopenias OR Low Bone Density OR Bone Density Low OR Low Bone Densities OR Low Bone Mineral Density) AND (Exercise OR Exercises OR Physical Activity OR Activities Physical OR Activity Physical OR Physical Activities OR Exercise Physical OR Exercises Physical OR Physical Exercise OR Physical Exercises OR Acute Exercise OR Acute Exercises OR Exercise Acute OR Exercises Acute OR Exercise Isometric OR Exercises Isometric OR Isometric Exercises OR Isometric Exercise OR Exercise Aerobic OR Aerobic Exercise OR Aerobic Exercises OR Exercises Aerobic OR Exercise Training OR Exercise Trainings OR Training Exercise OR Trainings Exercise) AND (anti resorptive therapy OR anti-resorptive therapies OR Therapy Drug OR Drug Therapies OR Therapies Drug OR Pharmacotherapy OR Pharmacotherapies) AND (Bone Biomarker OR Bone Biomarkers OR Remodeling Bone OR Bone Turnover OR Bone Turnovers OR Turnover Bone OR Turnovers Bone OR Bones and Bone Tissue OR Bones and Bone OR Bone Tissue OR Bone Tissues OR Tissue Bone OR Tissues Bone OR Bony Apophyses OR Apophyses Bony OR Bony Apophysis OR Apophysis Bony OR Condyle OR Condyles OR Bones OR Bone OR Bone Resorptions OR Resorption Bone OR Resorptions Bone OR Osteoclastic Bone Loss OR Bone Loss Osteoclastic OR Bone Losses Osteoclastic OR Loss Osteoclastic Bone OR Losses Osteoclastic Bone OR Osteoclastic Bone Losses OR Bone Formation OR Osteogenesis)).

Moreover, we conducted a grey literature search of other papers, using Proquest and Medrxiv, and hand searches of key conference proceedings, journals, professional organizations’ websites and guideline clearing houses. In accordance with the snowball technique, we examined references cited in the primary papers to identify additional papers.

### 2.2. Inclusion and Exclusion Criteria

The inclusion and exclusion criteria are described in Table 2. 

### 2.3. Data Extraction and Quality Assessment

On the basis of the above criteria (Table 2), reviewers screened the title and abstracts and selecting the eligible articles. Subsequently, potentially eligible full-text articles were downloaded and, after duplicates were removed, extracted and reviewed independently by the four reviewers (SM, AM, GB, YL) using a pre-tested data extraction form following the methods provided by the Cochrane Reviewers’ Handbook [43]. Finally, researchers extracted the data of the included studies. The details retrieved included: name of the first author, publication year, country, study design, population study with ages and number of experimental (EG) and control (CG) groups, sample size, type intensity and frequency of intervention, primary and secondary outcomes, results stratifying the studies for the different outcomes. Results were tabulated as mean ± SD where possible.

Any disagreement was settled by consensus (LD, LB, FM). When further information was needed, researchers contacted study authors by email [44].

Three researchers (SM, AM, GB) separately and blindly evaluated the selected studies for the risk of bias using the “Cochrane risk-of-bias tool for randomized trials” [45]. In order to resolve any quality score dispute, a fourth blind reviewer (YL) was involved as tiebreaker, when necessary. Risk of bias evaluation was made based on the primary outcome of our interest: bone turnover biomarkers, according to PRISMA guidelines [42].

The Cochrane risk-of-bias tool for randomized controlled trials analyses seven bias categories: (1) random sequence generation and (2) allocation concealment (regarding bias of selection and allocation), (3) selective reporting for reporting bias, (4) blinding of participants and personal (performance bias due to knowledge of the allocated intervention), (5) blinding of outcome assessment for detection bias, (6) incomplete outcomes data for bias in attrition, and another category (7) named “other bias” based on the probable bias not covered in the other domains. Each category results in a value of high, low or unclear (when the authors did not provide enough evidence about the bias category) risk of bias. We supplied a score to convert the Cochrane risk of bias tool to AHRQ (Agency for Healthcare Research and Quality) standards (Good, Fair and Poor).

## 3. Results

### 3.1. Study Selection and Characteristics

**Figure 1:**
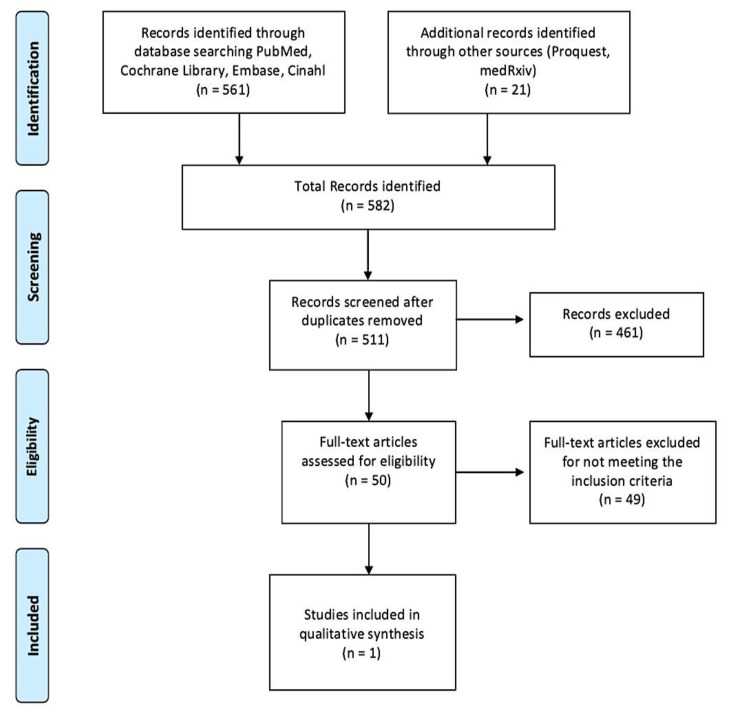
PRISMA flow diagram of the study selection.

Through databases browsed and hand search a total of 561 articles were identified (Figure 1). Additionally, 21 records were identified through other sources such as Proquest and medRxiv. As a result, the total number of records identified was 582. Papers were published from 2010 to 2020; 71 studies were excluded because duplicated, 461 studies were excluded following abstract and/or title review. Subsequently, we judged 50 records as relevant, 49 of which were subsequently excluded after a detailed full-text reading. The main causes of exclusion were related to the non-coherence with the aim of this study: the combined effect of pharmacological treatment and PA interventions on bone biomarkers in people with osteopenia and OP. In addition, most of the records (24%) were excluded due to the samples that did not match our inclusion criteria (population without primary OP). As a result, only one paper fully meeting the eligibility criteria, was finally included in the systematic review (Figure 1).

### 3.2. Risk of Bias

**Figure 2:**
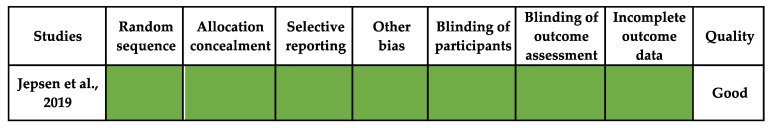
Quality assessment according to Cochrane Risk of Bias Tool for RCT. Note: green: criterion met, yellow: criterion unclear, red: criterion not met.

Randomized controlled trial’s quality was evaluated following the descriptive analysis. According to the Cochrane risk-of-bias tool for RCT we assessed the quality based on biomarkers outcome (Figure 2). The RCT explained in detail the computer-generated web-based block-randomization methods used to assign eligible participants to the intervention or control group (item #1) and allocation of participants after baseline evaluation (item #2). The selective reporting bias was assessed as clear due to availability of the study protocol previously registered (item#3).

Since the intervention consisted in PA there was no blinding of participants (item #4), however, considering primary outcome of the presents systematic review, we judged that the biomarkers outcome, is not likely to be influenced by lack of blinding of participants. Concerning the blinding of outcome assessment (item #5), Jepsen et al. described methods and techniques used to ensure the sensitivity of outcome assessment [46]. Regarding incomplete outcome data (item #6), we assessed low risk of bias because Jepsen et al. [46] reported all the outcomes data. Overall, the RCT included had no unclear criteria. Therefore, the risk of bias was scored as “Good quality”.

### 3.3. Data Extraction

Table 3 shows the main characteristics and results of the included study, evaluating the effects of whole-body vibration (WBV) combined with teriparatide on bone biomarkers, BMD and bone mineral content (BMC) in postmenopausal women with severe OP. All participants received subcutaneous teriparatide treatment (20 μg/day) and were advised to take supplements with calcium and vitamin D according to Danish osteoporosis treatment guidelines. The type of exercise training administered only to the experimental group was 12-min WBV training protocol previously tested in older population as safe, feasible and anabolic [47]. Since our aim was to assess the combined effects of pharmacological treatment and PA on bone biomarkers, we extracted the data considering the bone biomarkers analysis and other haematological parameters as primary outcome, whereas bone mineral density and bone microarchitecture assessment as secondary outcome. Regarding the bone biomarkers assessment, the study investigated the Procollagen type 1 N-terminal propeptide (P1NP), carboxy-terminal crosslinked telopeptide of type 1 collagen (CTX-1) which respectively reflect the anabolic and anti-resorptive metabolic effects and finally scerostin, a regulator of bone turnover.

Concerning the secondary outcomes, Jepsen et al. evaluated the response of bone mineral density estimated with DXA on the lumbar spine and total hip. Moreover, bone microarchitecture of non-dominant distal radius and tibia were included using HR-pQCT (XtremeCT; Scanco Medical, Zurich, Switzerland). After three and six months both groups showed a significant increase in CTX and P1NP but with no statically significant differences between groups. 

## 4. Discussion

Osteoporosis is a widespread bone illness considered as a more severe form of osteopenia. Nevertheless, osteopenia can be just as dangerous as OP especially when combined with other risk factors, such as smoking, a low-calcium diet, lack of vitamin D, hormonal changes due to age (especially menopause), and the presence of autoimmune conditions, such as rheumatoid arthritis [11]. The main consequences of these conditions are osteoporotic fractures, which lead to a substantial raise in mortality and morbidity of patients, with an enormous and heavy impact on both the quality of life of people and the economy of the health care system [48,49,50]. Moreover, pharmaco-economic considerations also play a role in this regard. [6]. Worse still, albeit the advances in pharmacotherapy, most of patients with osteopenia and OP are still not treated, and for whom begin to take medication, compliance to therapy is commonly below 50% at 1 to 2 years [51,52]. Furthermore, independently of bone tissue status, falls also represent a primary risk factor for osteoporotic fracture [53,54]. Certainly, anabolic and antiresorptive bone drugs increase BMD reducing fracture risk, yet without preventing falls [53]. Hence, in addition to pharmacological treatment, exercise can play a fundamental role since, alongside the strength effects on bone, it has the potential to improve muscle strength and balance, thus, also reducing the risk of falling [55]. Overall, the combined treatment of both pharmacological therapy and PA intervention might represent a viable strategy. In this context, bone turnover biomarkers can be evaluated to monitor the benefits of combined therapy in patients with OP or osteopenia.

Based on increasing professional and public awareness about chronic bone diseases, more scientific research to assess the combined treatment effects regarding OP or osteopenia, which are silently disabling and causing detrimental effects on all the life components, would be expected, yet to date there still a huge gap in this field. This is demonstrated by the fact that only one study, published in the last 10 years, has been included in our systematic review with the aim to investigate the effects of combined therapeutic strategy based on pharmacological treatment and PA interventions on bone biomarkers in people with osteopenia or OP. Although we are aware that very strict inclusion/exclusion criteria have been established for the present systematic review, this was suitably planned to avoid any possible misinterpretation of the results. Moreover, the very strict PICOST methodology used was crucial in guaranteeing the quality of the review. A case in point is represented by the years range established. Though some trials in early 2000s has been conducted in this regard, they could not be included in our systematic review, due to the study design used which did not met our inclusion criteria [56,57]. Surprisingly, one of the main reasons for the records exclusion was related to the fact that, although most of the studies were based on osteoporotic population samples, the absence of any pharmacological treatment stood out among the inclusion criteria. This could be partly due to the differences among countries in terms of healthcare systems and therapeutic strategies in this regard. Considering the existing evidence, it can be postulated that, although the guidelines for management and treatment of osteopenia and OP are widely accepted and recommended [58], there are certain difficulties associated with the implementation of pharmacological plans.

To note, in the RCT included in the review, the current standard recommended bone biomarkers CTX (resorption marker) and P1NP (formation marker) have been evaluated to investigate the combined effect of twelve-months WBV and teriparatide compared to teriparatide alone in postmenopausal women with severe OP. The findings have indicated an improvement during the study follow-up, but no additional effect of the combined treatment emerged. Interestingly, the authors have shown a similar improvement in BMD lumbar spine detected by DXA, which represents a secondary outcome result of our review. This can be considered an encouraging finding to understand the ability of bone biomarkers to monitor the effects of the strategies to treat OP and osteopenia.

Nowadays, OP has become a major public health issue worldwide given its healthcare cost, and there is a growing interest in multimodal care approaches including not only drug treatments but also exercise [58]. Indeed, PA interventions are a cost-effective, feasible and effective way to ameliorate these conditions, but to date, they are still not enough applied and evaluated [53]. Indeed, even though it is well known that exercise-induced mechanical load on the skeleton can improve bone strength by inducing adaptations in bone mass, a strong conclusion regarding the effects of exercise, in terms of intensity-duration-frequency of training, on bone tissue in osteoporotic people, has not yet been established [53]. Also, the only study included in our review, albeit of high quality, applied a combined intervention including teriparatide and WBV which envisages the use of a tool instead of a proper PA program [46]. Based on the available evidence analysed in the review, it seems that the combined strategies are scarcely applied to treat OP and osteopenia and worse still, poorly evaluated. Consistent with this, the challenge can be related to the limitations of currently available tools for monitoring these bone diseases.

A case in point is represented by bone turnover biomarkers. As already stated above in the “introduction” section, biomarkers are suitable tools to assess the dynamic metabolic bone status. Therefore, bone turnover biomarkers have become promising clinical parameters in the drug monitoring and management of OP [26]. In the meantime, data on the utility of biomarkers to assess the effects of exercise, alone or combined with drugs, on the bone tissue in patients with OP are still insufficient. In this scenario, our PICOST question might have been pretentious. However, we believe that our findings can be useful to highlight the gap in the literature, which needs to be bridged to understand the potential of biomarkers in the planning and evaluation of PA intervention for osteoporosis treatment. The identification of effective multiple intervention strategies can improve the quality of life of people affected by osteopenia and osteoporosis and reduce the burden of socio-economic costs associated with their treatment.

## 5. Conclusions

The possibility of estimating the effectiveness of therapeutic strategies aimed at treating OP is a pressing need for public health. Bone turnover biomarkers are being used for the diagnosis and management of OP and osteopenia. Our review aimed to evaluate the ability of bone biomarkers to assess not only the effects of drug therapy but also of PA interventions which are increasingly included as a positive additional strategy for the prevention and treatment of osteopenia and osteoporosis. Our analysis of the available literature has not reached a conclusion related to the review question. However, despite this, the article meeting the inclusion criteria was of high quality according to the Risk of Bias tool. For this reason, these findings may help to speculate considering bone biomarkers as a possible strategy to assess the combined effects of both drug therapy and PA treatments, albeit the result should be undoubtedly interpreted with caution. In addition, our review reveals the difficulty in assessing an interaction of drug therapy and PA, as most of the bone-target exercise intervention studies did not included patients taking pharmacological treatment. Future research is needed to fill this knowledge gap. The study of bone biomarkers to assess the effects of combined treatment (drug and PA) on bone tissue status is in its infancy. The study design of future RCTs should divide patients with OP or osteopenia into different experimental groups to evaluate the efficacy of the administration of drugs, PA or combined therapy. Moreover, in the planning of further studies the choice of the exercise training, in terms of intensity, duration and frequency, should have a key role, because the applied PA should be able to develop an adaptive response in bone. The availability of robust and clear evidence on applications of biomarkers will help healthcare professionals in their decision-making processes to plan interventions and guidelines for the treatment of OP and osteopenia. Overall, given the novel and unexplored features of this topic, as showed by the present systematic review, further investigations in this field are crucial to reduce the burden of socio-economic costs and to improve the health status and quality of life in people with osteopenia and OP.

## Figures and Tables

**Table 1:** Summary of pharmacological treatments currently available and their characteristics.

Drug Class	Mechanism of Action	Characteristics	References
**Anti-Resorptive**			
Biphosphonates	Interaction with specific intracellular pathways in osteoclasts, resulting in cellular toxicity	First-line pharmacological therapy for most post-menopausal women at risk for fractures. Efficacy in reducing the risk of vertebral, non-vertebral and hip	Pavone, 2017 [10]; Kanis, 2019 [13]; IOF, 2020 [14]; Compston, 2019 [15]
Denosumab	Inactivation of osteoclasts, reduction of osteoclasts’ differentiation	Strong efficacy in reducing spine and hip fractures. First-line therapy in patients intolerant to oral BP or having renal failure	Pavone, 2017 [10]; Kanis, 2019 [13]; Compston 2019 [15].
Menopausal Hormone Therapy /Hormone Replacement Therapy	Increase of bone mineral density at all skeletal sites in early and late postmenopausal women	Therapy to prevent bone loss and reduce fracture risk in women at high risk of fracture when alternate therapies are not appropriate	Pavone, 2017 [10]; Kanis, 2019 [13]; IOF, 2020 [14].
Selective Estrogen Receptor Modulators	Interaction with bone estrogen receptors, increasing trabecular bone mass	Contraindicated for prevention or treatment of OP in premenopausal women. Option treatment for younger postmenopausal women with osteopenia and osteoporosis without pronounced vasomotor menopausal symptoms, who are at risk for vertebral but not hip fractures	Pavone, 2017 [10]; Kanis, 2019 [13]; IOF, 2020 [14].
**Anabolic**			
Teriparatide	Activation of osteoblasts by binding the parathyroid hormone receptor; stimulation of bone formation on active remodelling sites, particularly in the trabecular compartment	Daily administration of subcutaneous injection for 18–24 months reduces the risk of vertebral and non-vertebral fracture in osteoporotic women	Pavone, 2017 [10]; Kanis, 2019 [13]; IOF, 2020 [14].
Abaloparatide	Selective activation of the parathyroid hormone receptor	Increase of BMD at the lumbar spine, femoral neck, and total hip; reduced risk of new vertebral fractures in postmenopausal patients with osteoporosis. It has been approved only in the USA	Compston, 2019 [15]; IOF, 2020 [14].
Romosozumab	Inhibition of sclerostin; Increase of bone formation and decrease of bone resorption	A starting treatment in women with high risk of fracture reduces the incidence of new vertebral fractures. The effects are reversible when the treatment is stopped, hence the therapy will need to be administered in sequence with an anti-resorptive drug	IOF, 2020 [14].

Note: BMD: Bone mineral density; OP: Osteoporosis.

**Table 2:** PICOST Eligibility criteria.

Parameter	Inclusion Criteria	Exclusion Criteria
Population	OP people (T-score ≤ 2.5) Osteopenic people (1 < T-score < 2.5) Aged 45–80+	Population with secondary OP Absence of OP diagnosis Different diseases
Intervention	PA combined with pharmacological treatment	Absence of PA and pharmacological treatment
Comparator	Standard pharmacological treatment No exercise intervention	Participants receiving different PA
Outcome	Bone biomarkers evaluation, physical performance or other indices of physical performance	No information about bone biomarkers and PA
Study design	Experimental or observational study with original primary data	Study Protocol or other papers without original data
Timing	English Language 10-year publication date limit (January 2011)	Not in English Language Published before January 2011

OP: osteoporosis PA: Physical activity.

**Table 3:** Study included in the review.

Study	Study Design	Sample	Intervention	Outcomes	Results
Jepsen et al., 2019, [46] Odense, Denmark	RCT	N:35 age:53–81 EG:17 CG:18	**Duration:** 12 weeks **Type of intervention:** EG: Whole-body vibration (WBV) training protocol 12 min × 3 session/week, rest ratio 1:1 min with a frequency of 30 Hz and amplitude of 1 mm + Teriparatide (20 μg/day) CG: Teriparatide (20 μg/day)	**Primary outcome:** CTX, P1NP Sclerostin **Secondary outcome:** BMD lumbar spine (L1-L4) and total hip, bone microarchitecture distal radius and tibia	**Primary outcomeresults** **Statistically significant improvement in CTX:** EG: *p* < 0.05 CG: *p* < 0.05 **Statistically significant improvement in P1NP:** EG: *p* < 0.05 CG: *p* < 0.05 **No statistically significant improvement in Sclerostin** **Secondary outcome results** **Statistically significant improvement in BMD lumbar spine of both group at 6 and 12 months** EG: 6.47% ± 3.40; 8.90% ± 5.48 CG: 3.48% ± 4.39; 6.65% ± 5.57 **No statistically significant improvement in BMD total hip** **No statistically significant improvement in bone microarchitecture**

## Data Availability

The data presented in the study are included in the article.
